# Supplementary material for: Oropouche virus cases identified in Ecuador using an optimised qRT-PCR informed by metagenomic sequencing
Source: PLoS Negl Trop Dis. 2020 Jan 21;14(1):e0007897. doi: 10.1371/journal.pntd.0007897 (PMC6994106; doi:10.1371/journal.pntd.0007897)
Supplement: S1 Text — (DOCX) [file pntd.0007897.s001.docx]

**S1 Text.** qRT-PCR assay optimisation and validation.

Primer (Table S1, reverse primer used was Ec2 R) concentrations were tested in multiple combinations at 1 µM, 3 µM, 9 µM and 18 µM. The optimal concentration for both the forward and reverse primer was 18 µM. Probe concentration was tested from 5 µM to 25 µM, with the optimal concentration being 12.5 µM. Magnesium sulphate (MgSO_4_) concentration was optimised by adding additional MgSO_4_ to the reaction mix, from none to a maximum of 85 mM. The optimal condition was no added MgSO_4_. Cross-reactivity to 23 virus species (Table S2) and a panel of negative human sera was assessed, no cross-reactions were observed.
