# Supplementary material for: Oropouche virus cases identified in Ecuador using an optimised qRT-PCR informed by metagenomic sequencing
Source: PLoS Negl Trop Dis. 2020 Jan 21;14(1):e0007897. doi: 10.1371/journal.pntd.0007897 (PMC6994106; doi:10.1371/journal.pntd.0007897)
Supplement: S2 Text — (DOCX) [file pntd.0007897.s002.docx]

**S2 Text.** Multiplex tiling PCR primer details.

Primer details are available as a .csv file at the following link:

[Supplementary material. OROV multiplex tiling PCR primers](https://drive.google.com/open?id=1yM8tBnvOBzlWjWcv8LYoAwPmkr5nCNSt)
