# Supplementary material for: Oropouche virus cases identified in Ecuador using an optimised qRT-PCR informed by metagenomic sequencing
Source: PLoS Negl Trop Dis. 2020 Jan 21;14(1):e0007897. doi: 10.1371/journal.pntd.0007897 (PMC6994106; doi:10.1371/journal.pntd.0007897)
Supplement: S1 Table — (DOCX) [file pntd.0007897.s003.docx]

| **Oligo name** | **Sequence (5' - 3')** | **Start position** | **End position** | **Length (bp)** | **Tm (°C)** | **GC content (%)** | **Reference** |
| --- | --- | --- | --- | --- | --- | --- | --- |
| OROV F | CATTTGAAGCTAGATACGGACAA | 118 | 140 | 23 | 59 | 39 | Weidmann *et al.* 2003 |
| OROV R | CCATGGGCCTCGATG | 225 | 211 | 15 | 52 | 67 | Weidmann *et al.* 2003 |
| Ec R | CCATGGGCCGCGACG | 225 | 211 | 15 | 57 | 80 | This study |
| Ec2 R | CATCTTTGGCCTTCTTTTRG | 198 | 179 | 20 | 54-56 | 40-45 | This study |
| OROV P | CAATGCTGGTGTTGTTAGAGTCTTCTTCCT | 146 | 175 | 30 | 69 | 43 | Weidmann *et al.* 2003 |

**S1 Table.** Oligonucleotides used in the development of the OROV qRT-PCR.

**References**

[Weidmann M, Rudaz V, Nunes MRT, Vasconcelos PFC, Hufert FT. Rapid detection of human pathogenic orthobunyaviruses. J Clin Microbiol. 2003 Jul;41(7):3299–305.](http://paperpile.com/b/Z4MJR3/Kdlg)
