# Supplementary material for: Oropouche virus cases identified in Ecuador using an optimised qRT-PCR informed by metagenomic sequencing
Source: PLoS Negl Trop Dis. 2020 Jan 21;14(1):e0007897. doi: 10.1371/journal.pntd.0007897 (PMC6994106; doi:10.1371/journal.pntd.0007897)
Supplement: S2 Table — (DOCX) [file pntd.0007897.s004.docx]

| **Virus family** | **Virus genus** | **Virus species** | **Virus acronym** |
| --- | --- | --- | --- |
| Arenaviridae | Mammarenavirus | Tamiami mammarenavirus | TAMV |
| Flaviviridae | Flavivirus | Powassan virus | POWV |
| Flaviviridae | Flavivirus | West Nile virus | WNV |
| Flaviviridae | Flavivirus | Yellow fever virus | YFV |
| Flaviviridae | Flavivirus | Karshi virus | KSIV |
| Flaviviridae | Flavivirus | Usutu virus | USUV |
| Flaviviridae | Flavivirus | Dengue virus serotype 1 | DENV-1 |
| Flaviviridae | Flavivirus | Dengue virus serotype 2 | DENV-2 |
| Flaviviridae | Flavivirus | Dengue virus serotype 3 | DENV-3 |
| Flaviviridae | Flavivirus | Dengue virus serotype 4 | DENV-4 |
| Flaviviridae | Flavivirus | Zika virus | ZIKV |
| Nairoviridae | Orthonairovirus | Crimean-Congo hemorrhagic fever orthonairovirus | CCHFV |
| Nairoviridae | Orthonairovirus | Issyk-Kul virus | ISKV |
| Peribunyaviridae | Orthobunyavirus | Batai orthobunyavirus | BATV |
| Peribunyaviridae | Orthobunyavirus | La Crosse orthobunyavirus | LACV |
| Peribunyaviridae | Orthobunyavirus | Inkoo virus | INKV |
| Peribunyaviridae | Orthobunyavirus | Tahyna virus | TAHV |
| Phenuiviridae | Phlebovirus | Bhanja virus | BHAV |
| Phenuiviridae | Phlebovirus | Severe fever with thrombocytopenia syndrome  virus | SFTSV |
| Phenuiviridae | Phlebovirus | Rift Valley fever phlebovirus | RVFV |
| Togaviridae | Alphavirus | Chikungunya virus | CHIKV |
| Togaviridae | Alphavirus | Mayaro virus | MAYV |
| Togaviridae | Alphavirus | O'nyong-nyong virus | ONNV |

**S2 Table.** Viruses tested for cross-reactivity with the qRT-PCR.
