# Supplementary material for: Oropouche virus cases identified in Ecuador using an optimised qRT-PCR informed by metagenomic sequencing
Source: PLoS Negl Trop Dis. 2020 Jan 21;14(1):e0007897. doi: 10.1371/journal.pntd.0007897 (PMC6994106; doi:10.1371/journal.pntd.0007897)
Supplement: S3 Table — Values are the number of sequences with mismatches to the primer/probe sequence. (DOCX) [file pntd.0007897.s005.docx]

| **Number of mismatches** | **Forward primer** | **Reverse primer (OROV R)** | **Reverse primer**  **(Ec2 R)** | **Probe** |
| --- | --- | --- | --- | --- |
| **1** | 17 | 20 | 41 | 21 |
| **2** | 2 | 6 | 0 | 0 |
| **>2** | 0 | 0 | 0 | 0 |

**S3 Table.** Mismatches to oligonucleotide sequences observed in an alignment of 149 OROV N gene sequences. Values are the number of sequences with mismatches to the primer/probe sequence.
