# Supplementary material for: Oropouche virus cases identified in Ecuador using an optimised qRT-PCR informed by metagenomic sequencing
Source: PLoS Negl Trop Dis. 2020 Jan 21;14(1):e0007897. doi: 10.1371/journal.pntd.0007897 (PMC6994106; doi:10.1371/journal.pntd.0007897)
Supplement: S4 Table — Genome copies/ mL plasma are estimated based on the absolute quantitation standard curve. nd = no data. (DOCX) [file pntd.0007897.s006.docx]

| **Sample ID** | **Sex (M/F)** | **Age** | **Days of Fever** | **OROV qRT-PCR (Cq)** | **Estimated genome copies/ml plasma** |
| --- | --- | --- | --- | --- | --- |
| D-057 | M | 35 | 3 | 25.66 | 1.26 x 10^9^ |
| D-087 | M | 41 | 7 | 36.26 | 9.62 x 10^3^ |
| D-155 | M | nd | 2 | 25.75 | 1.19 x 10^9^ |
| D-171 | F | nd | 2 | 26.75 | 6.04 x 10^8^ |
| D-206 | M | nd | 4 | 30.87 | 1.97 x 10^7^ |
| D-210 | M | nd | 3 | 29.18 | 9.27 x 10^7^ |

**S4 Table.** Ecuadorian OROV-positive patient samples, determined by OROV S segment qRT-PCR. Genome copies/ mL plasma are estimated based on the absolute quantitation standard curve. nd = no data.
