# Supplementary material for: Oropouche virus cases identified in Ecuador using an optimised qRT-PCR informed by metagenomic sequencing
Source: PLoS Negl Trop Dis. 2020 Jan 21;14(1):e0007897. doi: 10.1371/journal.pntd.0007897 (PMC6994106; doi:10.1371/journal.pntd.0007897)
Supplement: S5 Table — Variant base is shaded grey. * R position = 79% T, 21% C. (DOCX) [file pntd.0007897.s007.docx]

| Genome position | Genome segment | Gene | Position within gene | D-057 base | D-087 base | D-155 base | D-171 base | D-206 base | D-210 base |
| --- | --- | --- | --- | --- | --- | --- | --- | --- | --- |
| 329 | S | N | 285 | G | G | G | G | A | G |
| 551 | S | N | 507 | T | C | C | C | C | C |
| 689 | S | N | 645 | A | G | G | G | G | G |
| 1501 | M | M | 518 | A | A | T | A | A | A |
| 1751 | M | M | 768 | T | T | T | T | C | T |
| 2038 | M | M | 1055 | C | C | C | C | T | C |
| 2230 | M | M | 1247 | A | A | A | A | A | G |
| 2363 | M | M | 1380 | T | C | T | T | T | T |
| 2403 | M | M | 1420 | A | G | G | G | G | G |
| 2810 | M | M | 1827 | G | R* | G | G | G | G |
| 2859 | M | M | 1876 | A | A | A | A | A | G |
| 3290 | M | M | 2307 | T | T | C | T | T | T |
| 4028 | M | M | 3045 | C | C | C | C | C | A |
| 4124 | M | M | 3141 | G | G | A | G | G | G |
| 4313 | M | M | 3330 | T | T | T | T | C | T |
| 4340 | M | M | 3357 | G | A | A | A | A | A |
| 4490 | M | M | 3507 | T | C | C | C | C | C |
| 6129 | L | L | 747 | T | C | T | T | T | T |
| 6174 | L | L | 792 | A | A | A | A | G | A |
| 6200 | L | L | 818 | A | A | A | A | A | G |
| 6579 | L | L | 1197 | G | G | G | G | A | G |
| 7599 | L | L | 2217 | T | C | C | C | C | C |
| 8865 | L | L | 3483 | G | A | A | A | A | A |
| 9235 | L | L | 3853 | T | T | T | T | T | C |
| 9336 | L | L | 3954 | G | G | A | G | G | G |
| 9571 | L | L | 4189 | A | A | G | A | A | A |
| 9591 | L | L | 4209 | G | A | G | G | G | G |
| 10039 | L | L | 4657 | A | G | G | G | G | G |
| 10737 | L | L | 5355 | A | A | A | G | A | A |
| 10791 | L | L | 5409 | C | C | C | C | C | T |
| 11133 | L | L | 5751 | C | T | C | C | C | C |
| 11208 | L | L | 5826 | C | C | C | C | A | C |
| 11733 | L | L | 6351 | C | C | C | C | T | C |

**S5 Table.** SNPs identified between six Ecuadorian OROV genomes (sequenced from P1 Vero cell supernatant). Variant base is shaded grey. * R position = 79% T, 21% C.
