# Supplementary material for: Oropouche virus cases identified in Ecuador using an optimised qRT-PCR informed by metagenomic sequencing
Source: PLoS Negl Trop Dis. 2020 Jan 21;14(1):e0007897. doi: 10.1371/journal.pntd.0007897 (PMC6994106; doi:10.1371/journal.pntd.0007897)
Supplement: S6 Table — AA = amino acid. Gn = glycoprotein Gn. NSm = non-structural protein NSm. Gc = glycoprotein Gc. Bunyavirus Gn, NSm and Gc protein positions are taken from GenPept entry AGH07923.1. R group = reactive group. (DOCX) [file pntd.0007897.s008.docx]

| **Protein** | **Isolate** | **Codon** | **Consensus AA** | **SNP AA** | **R group change** |
| --- | --- | --- | --- | --- | --- |
| NSs | D-206 | 88 | C | Y | None |
| M (Gn) | D-155 | 173 | Q | L | Polar / non-polar |
| M (NSm) | D-206 | 352 | A | V | None |
| M (NSm) | D-210 | 416 | K | R | None |
| M (NSm) | D-057 | 464 | A | T | Non-polar / polar |
| M (Gc) | D-210 | 626 | T | A | Polar / non-polar |
| L | D-210 | 273 | D | G | Acidic / non-polar |
| L | D-155 | 1397 | T | A | Polar / non-polar |

**S6 Table.** Amino acid variation between six Ecuadorian OROV genomes. AA = amino acid. Gn = glycoprotein Gn. NSm = non-structural protein NSm. Gc = glycoprotein Gc. Bunyavirus Gn, NSm and Gc protein positions are taken from GenPept entry AGH07923.1. R group = reactive group.
