# Supplementary material for: Oropouche virus cases identified in Ecuador using an optimised qRT-PCR informed by metagenomic sequencing
Source: PLoS Negl Trop Dis. 2020 Jan 21;14(1):e0007897. doi: 10.1371/journal.pntd.0007897 (PMC6994106; doi:10.1371/journal.pntd.0007897)
Supplement: S7 Table — n/a = no patient genome data available. (DOCX) [file pntd.0007897.s009.docx]

| **OROV strain** | **S** | **M** | **L** | **Total** |
| --- | --- | --- | --- | --- |
| D-057 | 1 | 0 | 0 | 1 |
| D-087 | 1 | 6 | 5 | 12 |
| D-155 | 2 | 6 | 7 | 15 |
| D-171 | 1 | 2 | 3 | 6 |
| D-206 | nd | 2 | 7 | 9 |
| D-210 | 0 | 2 | 5 | 7 |

**S7 Table.** A summary of the number of SNPs present in each OROV genome segment (S, M and L), between patient and cultured genome sequences. n/a = no patient genome data available.
