# Supplementary material for: Oropouche virus cases identified in Ecuador using an optimised qRT-PCR informed by metagenomic sequencing
Source: PLoS Negl Trop Dis. 2020 Jan 21;14(1):e0007897. doi: 10.1371/journal.pntd.0007897 (PMC6994106; doi:10.1371/journal.pntd.0007897)
Supplement: S8 Table — Variance within each genome is also shown as the percentage of reads at that position showing a particular base. Seg. = segment. Seg. pos. = segment position. Cons. = consensus. (DOCX) [file pntd.0007897.s010.docx]

| Isolate | Seg. | Seg. pos. | Patient cons. | Depth | A (%) | C (%) | G (%) | T (%) | Culture cons. | Depth | A (%) | C (%) | G (%) | T (%) |
| --- | --- | --- | --- | --- | --- | --- | --- | --- | --- | --- | --- | --- | --- | --- |
| D-057 | M | 59 | W | 21 | 23.8 | 0.0 | 0.0 | 76.2 | A | 405 | 95.1 | 0.0 | 0.0 | 0.0 |
| D-087 | S | 300 | T | 16 | 0.0 | 0.0 | 0.0 | 100.0 | C | 52430 | 0.0 | 99.9 | 0.0 | 0.0 |
| D-087 | M | 59 | T | 23 | 0.0 | 0.0 | 0.0 | 100.0 | A | 85 | 96.5 | 0.0 | 0.0 | 0.0 |
| D-087 | M | 307 | Y | 1055 | 0.0 | 23.7 | 0.0 | 76.3 | T | 2419 | 0.0 | 0.0 | 0.0 | 99.8 |
| D-087 | M | 1565 | Y | 286 | 0.0 | 21.0 | 0.0 | 79.0 | T | 5365 | 0.0 | 0.0 | 0.0 | 99.8 |
| D-087 | M | 1858 | G | 129 | 0.0 | 0.0 | 100.0 | 0.0 | R | 5135 | 55.5 | 0.0 | 44.4 | 0.0 |
| D-087 | M | 3097 | M | 55 | 29.1 | 70.9 | 0.0 | 0.0 | A | 6677 | 99.7 | 0.0 | 0.0 | 0.0 |
| D-087 | M | 3650 | G | 28 | 0.0 | 0.0 | 100.0 | 0.0 | A | 3710 | 99.8 | 0.0 | 0.0 | 0.0 |
| D-087 | L | 122 | G | 8 | 0.0 | 0.0 | 100.0 | 0.0 | C | 2575 | 0.0 | 98.6 | 0.0 | 1.2 |
| D-087 | L | 312 | R | 33 | 75.8 | 0.0 | 24.2 | 0.0 | A | 9936 | 99.8 | 0.0 | 0.0 | 0.0 |
| D-087 | L | 1855 | R | 56 | 25.0 | 0.0 | 75.0 | 0.0 | A | 3997 | 99.4 | 0.0 | 0.0 | 0.0 |
| D-087 | L | 3114 | A | 315 | 99.7 | 0.0 | 0.0 | 0.0 | C | 1923 | 0.0 | 99.9 | 0.0 | 0.0 |
| D-087 | L | 3875 | T | 775 | 0.0 | 1.4 | 0.0 | 98.6 | C | 11091 | 0.0 | 99.9 | 0.0 | 0.0 |
| D-155 | S | 107 | G | 19 | 5.3 | 0.0 | 94.7 | 0.0 | A | 4060 | 98.8 | 0.0 | 0.0 | 0.0 |
| D-155 | S | 880 | C | 6 | 0.0 | 100.0 | 0.0 | 0.0 | T | 14890 | 0.0 | 0.0 | 0.0 | 99.7 |
| D-155 | M | 160 | G | 17 | 17.6 | 0.0 | 82.4 | 0.0 | A | 10115 | 92.9 | 0.0 | 6.7 | 0.0 |
| D-155 | M | 1586 | K | 35 | 0.0 | 0.0 | 22.9 | 77.1 | G | 49818 | 0.0 | 0.0 | 99.7 | 0.0 |
| D-155 | M | 2191 | R | 14 | 71.4 | 0.0 | 28.6 | 0.0 | G | 46084 | 0.0 | 0.0 | 100.0 | 0.0 |
| D-155 | M | 2821 | W | 729 | 30.0 | 0.0 | 0.0 | 70.0 | T | 59384 | 0.0 | 0.0 | 0.0 | 99.9 |
| D-155 | M | 2931 | M | 370 | 23.2 | 76.8 | 0.0 | 0.0 | C | 55496 | 0.0 | 99.8 | 0.0 | 0.0 |
| D-155 | M | 4033 | C | 123 | 0.0 | 98.4 | 1.6 | 0.0 | G | 8704 | 0.0 | 0.0 | 99.9 | 0.0 |
| D-155 | L | 6 | A | 110 | 100.0 | 0.0 | 0.0 | 0.0 | T | 587 | 0.0 | 0.0 | 0.0 | 98.3 |
| D-155 | L | 1483 | R | 10 | 70.0 | 0.0 | 30.0 | 0.0 | A | 49020 | 100.0 | 0.0 | 0.0 | 0.0 |
| D-155 | L | 1664 | R | 185 | 65.4 | 0.0 | 34.6 | 0.0 | A | 45106 | 99.7 | 0.0 | 0.0 | 0.0 |
| D-155 | L | 2219 | R | 119 | 73.9 | 0.0 | 26.1 | 0.0 | A | 13427 | 99.9 | 0.0 | 0.0 | 0.0 |
| D-155 | L | 3321 | S | 57 | 0.0 | 45.6 | 54.4 | 0.0 | C | 19968 | 0.0 | 99.9 | 0.0 | 0.0 |
| D-155 | L | 5126 | K | 4019 | 0.0 | 0.0 | 26.5 | 73.5 | G | 23406 | 0.0 | 0.0 | 99.9 | 0.0 |
| D-155 | L | 6206 | R | 572 | 75.0 | 0.0 | 24.8 | 0.0 | A | 8425 | 99.5 | 0.0 | 0.0 | 0.0 |
| D-171 | S | 41 | G | 5 | 0.0 | 0.0 | 100.0 | 0.0 | A | 1008 | 97.7 | 0.0 | 0.0 | 0.0 |
| D-171 | M | 144 | C | 71 | 0.0 | 95.8 | 0.0 | 0.0 | T | 6256 | 0.0 | 0.0 | 0.0 | 99.8 |
| D-171 | M | 1940 | Y | 2955 | 0.0 | 57.5 | 0.0 | 42.5 | T | 86762 | 0.0 | 0.0 | 0.0 | 99.6 |
| D-171 | L | 5165 | Y | 1251 | 0.0 | 24.7 | 0.0 | 75.3 | T | 34965 | 0.0 | 0.0 | 0.0 | 99.7 |
| D-171 | L | 6298 | Y | 483 | 0.0 | 70.6 | 0.0 | 29.4 | T | 7615 | 0.0 | 0.0 | 0.0 | 99.4 |
| D-171 | L | 6308 | M | 61 | 44.3 | 55.7 | 0.0 | 0.0 | A | 7409 | 99.6 | 0.0 | 0.0 | 0.0 |
| D-206 | M | 1060 | T | 9 | 0.0 | 0.0 | 0.0 | 88.9 | A | 43090 | 99.8 | 0.0 | 0.0 | 0.0 |
| D-206 | M | 3968 | C | 43 | 0.0 | 100.0 | 0.0 | 0.0 | T | 15874 | 0.0 | 0.0 | 0.0 | 99.6 |
| D-206 | L | 208 | G | 109 | 0.0 | 0.0 | 100.0 | 0.0 | T | 21634 | 0.0 | 0.0 | 0.0 | 99.1 |
| D-206 | L | 222 | Y | 2013 | 0.0 | 69.0 | 0.0 | 30.9 | C | 35017 | 0.0 | 98.4 | 0.0 | 0.0 |
| D-206 | L | 223 | Y | 2040 | 0.0 | 31.2 | 0.0 | 68.8 | T | 35739 | 0.0 | 1.8 | 0.0 | 97.0 |
| D-206 | L | 1041 | R | 8270 | 21.0 | 0.0 | 79.0 | 0.0 | G | 48064 | 0.0 | 0.0 | 99.6 | 0.0 |
| D-206 | L | 2143 | S | 1024 | 0.0 | 63.8 | 36.1 | 0.0 | C | 15102 | 0.0 | 99.7 | 0.0 | 0.0 |
| D-206 | L | 4093 | R | 368 | 75.8 | 0.0 | 24.2 | 0.0 | A | 27954 | 99.7 | 0.0 | 0.0 | 0.0 |
| D-206 | L | 4381 | S | 110 | 0.0 | 66.4 | 33.6 | 0.0 | G | 30517 | 0.0 | 0.0 | 99.9 | 0.0 |
| D-210 | M | 3929 | Y | 5066 | 0.0 | 53.2 | 0.0 | 46.8 | C | 17041 | 0.0 | 100.0 | 0.0 | 0.0 |
| D-210 | M | 4376 | W | 230 | 53.0 | 0.0 | 0.0 | 46.5 | T | 365 | 0.0 | 0.0 | 0.0 | 99.5 |
| D-210 | L | 91 | T | 49 | 0.0 | 0.0 | 0.0 | 98.0 | C | 5779 | 0.0 | 99.7 | 0.0 | 0.0 |
| D-210 | L | 217 | W | 4454 | 29.8 | 0.0 | 0.0 | 68.4 | T | 25397 | 0.0 | 1.3 | 0.0 | 94.0 |
| D-210 | L | 3238 | R | 4655 | 70.3 | 0.0 | 29.7 | 0.0 | A | 26664 | 99.8 | 0.0 | 0.0 | 0.0 |
| D-210 | L | 4111 | R | 466 | 36.3 | 0.0 | 63.7 | 0.0 | G | 29698 | 0.0 | 0.0 | 99.9 | 0.0 |
| D-210 | L | 5585 | G | 967 | 2.5 | 0.0 | 97.5 | 0.0 | A | 20436 | 99.8 | 0.0 | 0.0 | 0.0 |

**S8 Table.** Positions in the OROV genome at which SNPs were identified between the patient and cultured genome, for each isolate. Variance within each genome is also shown as the percentage of reads at that position showing a particular base. Seg. = segment. Seg. pos. = segment position. Cons. = consensus.
