# Supplementary material for: Oropouche virus cases identified in Ecuador using an optimised qRT-PCR informed by metagenomic sequencing
Source: PLoS Negl Trop Dis. 2020 Jan 21;14(1):e0007897. doi: 10.1371/journal.pntd.0007897 (PMC6994106; doi:10.1371/journal.pntd.0007897)
Supplement: S1 Fig — (DOCX) [file pntd.0007897.s011.docx]

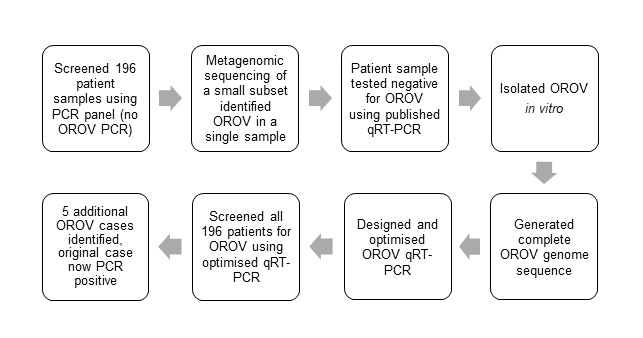


**S1 Figure.** The workflow that led to the identification and isolation of OROV from six febrile Ecuadorian patients.
