# Supplementary material for: Oropouche virus cases identified in Ecuador using an optimised qRT-PCR informed by metagenomic sequencing
Source: PLoS Negl Trop Dis. 2020 Jan 21;14(1):e0007897. doi: 10.1371/journal.pntd.0007897 (PMC6994106; doi:10.1371/journal.pntd.0007897)
Supplement: S2 Fig — Each data point is the mean Cq value from three separate experiments. Error bars indicate standard deviation. R2 correlation coefficient = 0.9978. (DOCX) [file pntd.0007897.s012.docx]

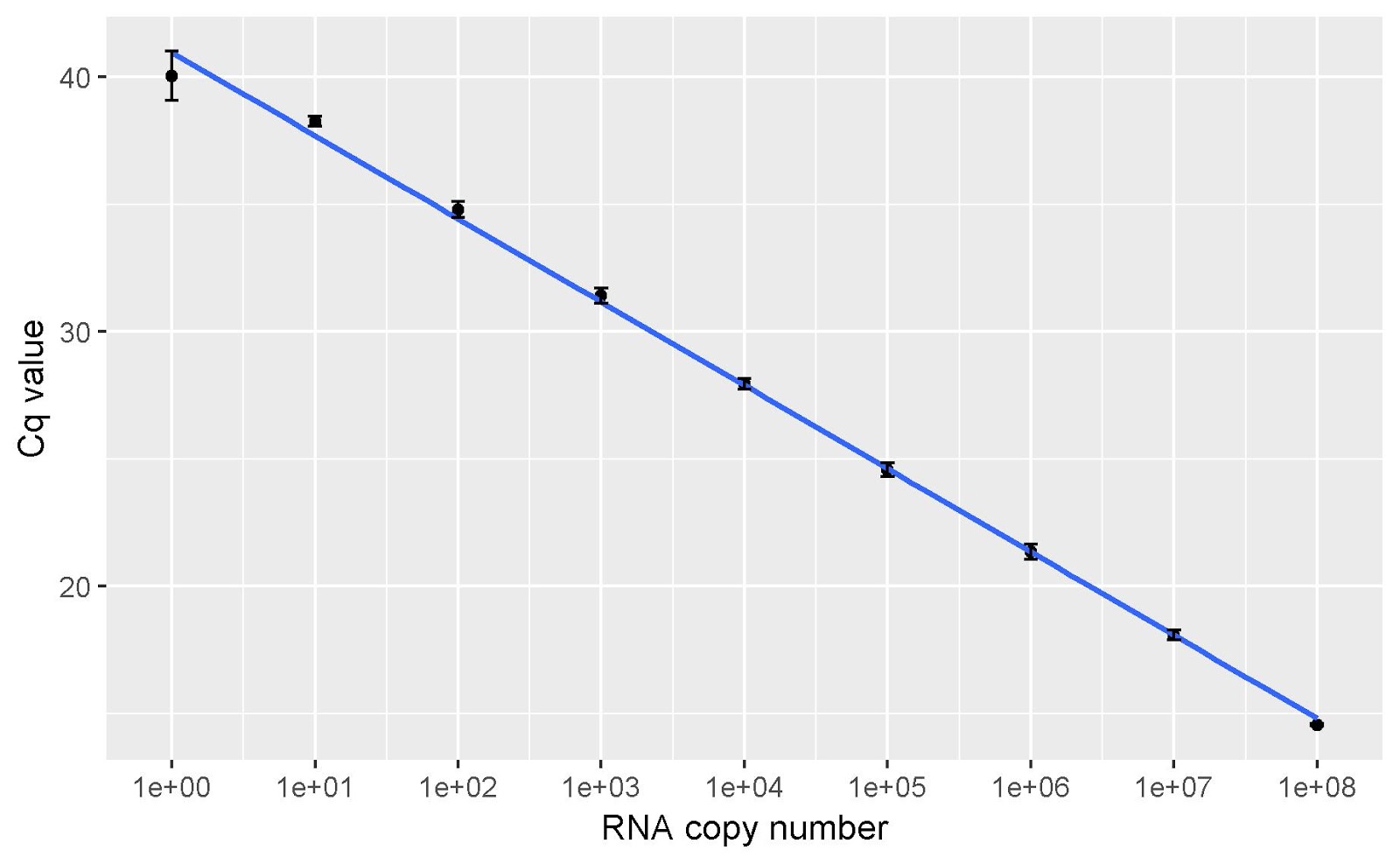


**S2 Figure.** Absolute quantitation was performed from a standard curve generated from a ten-fold serial dilution of a synthetic OROV RNA standard. Each data point is the mean Cq value from three separate experiments. Error bars indicate standard deviation. R^2^ correlation coefficient = 0.9978.
