# Supplementary material for: Oropouche virus cases identified in Ecuador using an optimised qRT-PCR informed by metagenomic sequencing
Source: PLoS Negl Trop Dis. 2020 Jan 21;14(1):e0007897. doi: 10.1371/journal.pntd.0007897 (PMC6994106; doi:10.1371/journal.pntd.0007897)
Supplement: S3 Fig — (DOCX) [file pntd.0007897.s013.docx]

**
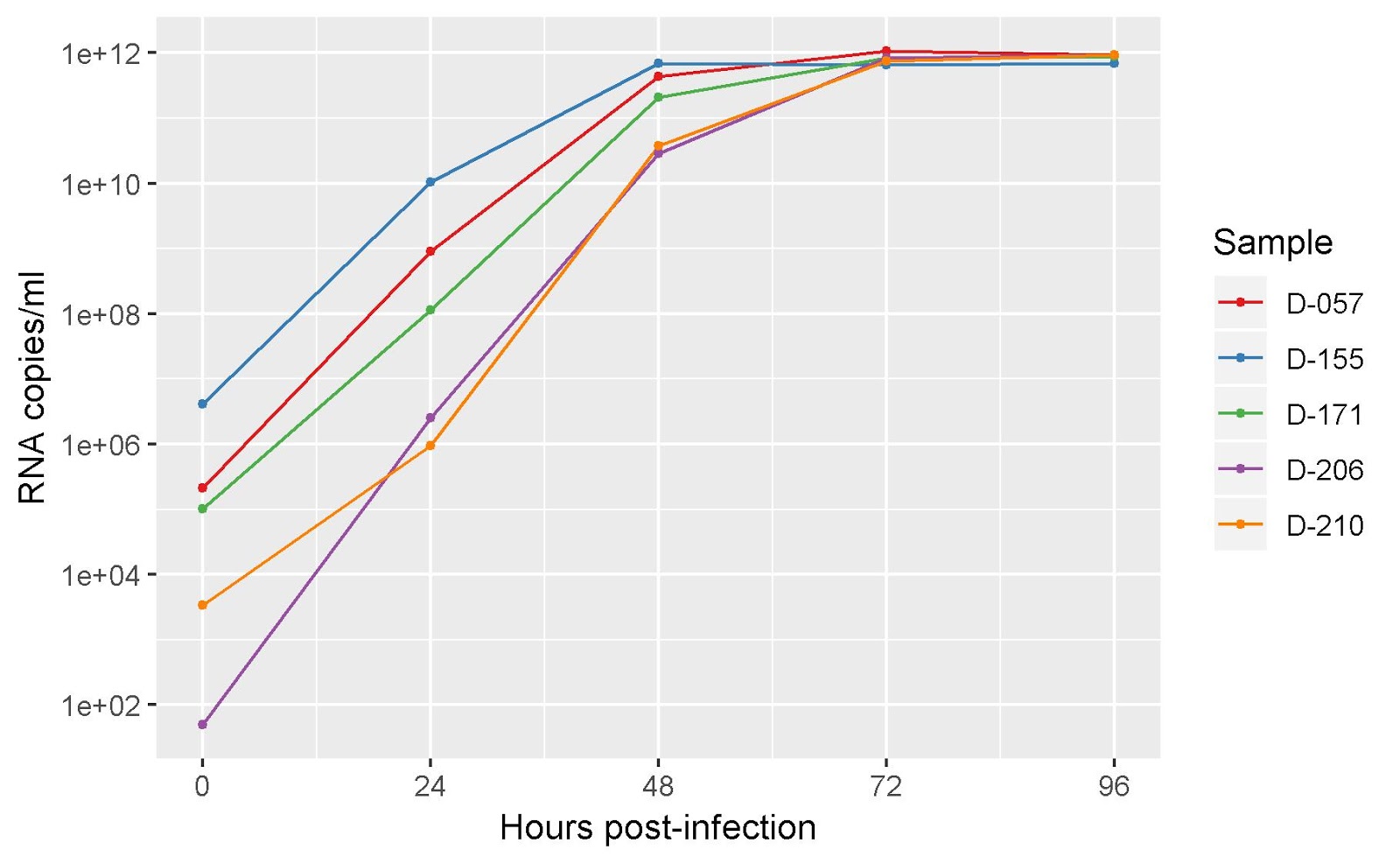
**

**S3 Figure.** OROV genome copies increase over 96 hours in Vero cells, demonstrating OROV genome replication in five independent OROV cultures from OROV-positive patient plasma.
